# Do Animations Impair Executive Function in Young Children? Effects of Animation Types on the Executive Function of Children Aged Four to Seven Years

**DOI:** 10.3390/ijerph19158962

**Published:** 2022-07-23

**Authors:** Liheng Fan, Meng Lu, Xiuxiu Qi, Jie Xin

**Affiliations:** 1 Institute of Psychology and Behavior, Henan University, Kaifeng 475004, China; fanliheng@163.com; 2 Faculty of Education, Henan University, Kaifeng 475004, China; 15225275755@163.com (X.Q.); xinj77@163.com (J.X.); 3 Graduate School of Manga, Kyoto Seika University, Kyoto 606-8588, Japan

**Keywords:** educational animation, entertainment animation, executive function, short-term effect, children aged four to seven years

## Abstract

This study used a three (animation types: educational, entertainment, and control groups) × four (age group: four-, five-, six-, and seven-year-olds) between-group experimental design to investigate the short-term effects of animation type and age on each component of children’s executive function (EF) (inhibitory control [IC], cognitive flexibility [CF], and working memory [WM]). One hundred twenty-six kindergarten and first-grade elementary school students in a city in Henan Province of China were selected for the experimental study. The results showed that briefly watching animation affected children’s EF. Specifically, watching entertainment cartoons weakened children’s IC and CF, while cartoons did not affect children’s WM. The moderating effect of age in the relationship between animation type and EFs was non-significant. This study suggests that researchers should focus on the uniqueness of each component of EF in children aged four to seven years, and parents should try to limit children’s viewing of animation, especially entertainment animation.

## 1. Introduction

Executive function (EF) is the ability of individuals to use thinking skills to achieve goals, develop a set of problem-solving methods, and monitor and adjust their behavior. EF is a higher-level processing activity of the brain. The process allows individuals to exercise conscious control over their thoughts and actions [1]. EF generally includes inhibitory control (IC), working memory (WM), and cognitive flexibility (CF) [2]. In addition, EF in early childhood can effectively predict an individual’s behavior [3,4,5]. Therefore, studying the influences of EF is essential to understanding and improving people’s daily learning, work, and life.

### 1.1. Television and EF

The influence of the environment becomes significant in early childhood when the brain rapidly develops strong neuroplasticity [6]. Television viewing is a ubiquitous behavior for children, with many beginning to watch television in preschool or as early as infancy [7]. Data from a large sample of approximately 9000 children in the US found that children watched approximately four hours of television per day [8]. Chinese data on over 1000 young children showed that the proportion of children under two years of age watching television was 58.7%, rising to 93.2% between two and four years of age. On average, 19.9% of children aged two to four watch more than two hours daily [9]. Considering the critical influence of the environment on the development of EF in early childhood and that television exposure serves as one of the primary environments in early childhood, television exposure may play an essential role in the development of EF in early childhood. Therefore, many researchers have begun to explore the relationship between the two.

Earlier studies on television exposure have mainly concentrated on its relationship with attention-related variables. For instance, Christakis et al. found that the amount of time spent watching television in early childhood positively predicted attention problems in middle childhood [10]. Miller et al. also found a significant positive association between television exposure time in preschoolers and their attention deficit and hyperactivity tendencies [11]. Further, Nathanson et al. examined the effects of viewing time and background time on EF in young children. They found a negative association between television viewing and background television exposure to EF in young children [12]. However, some studies did not find a relationship between television exposure and attention problems [13,14]. For example, Stevens and Mulsow found that the effect size associated with television viewing time and later attention problems in preschoolers was close to zero [15].

In response to these inconsistent results, some researchers have suggested that television content may play a more critical role in children’s EF development than television time [16,17]. As a result, researchers have begun to focus on the effects of educational and entertainment television programs on children’s EF and other cognitive functions.

Some studies found that viewing entertainment-based programs resulted in young children showing poorer cognitive responses than viewing educational programs [18,19]. An experimental study by Li on preschoolers aged three to six also found that children who watched entertainment programs for a short period had significantly lower EF scores than children in the educational and control groups [20]. In addition, Yang et al. used a questionnaire to explore the relationship between different animation types and children’s EF. They found a critical positive association between educational animation and preschoolers’ EF [21]. However, some studies have found different results. For example, Lillard et al. had four-year-old children watch entertainment animation, watch educational animation, or perform autonomous drawing (control group) and then tested subsequent performance on EF tasks. They found that the EF scores of the animation-viewing group were lower than those of the control group, but the differences between animation groups were non-significant. In other words, the EF performance of four-year-old children temporarily diminished when watching entertainment or educational animation [22]. Moreover, Zimmerman and Christakis examined the relationship between three types of animated programs—educational, nonviolent, and violent entertainment—and attention problems. They found that viewing educational programs before the age of three years was not associated with attention problems five years later, whereas viewing violent and nonviolent entertainment programs before that age was significantly associated with attention problems five years later [23].

### 1.2. The Current Study

Are there short-term effects of animation type on EF in young children? Current studies have not been able to produce consistent results. Previous studies mainly focused on the impact on the overall level of EF while ignoring issues such as the independence and unique impact of each component of EF. In addition, several studies have found that different components of EF have different developmental trajectories and prefrontal cortex localization [24,25,26,27]. Therefore, understanding the specific effects of animation type on each component of EF in children is vital to clarify research findings, guide future research directions, and provide practical interventions. We selected educational and entertainment animations and used drawing as a control group to answer these questions. The study explored whether viewing different types of cartoons affects children’s EF.

The Differential Susceptibility to Media Effects Model suggests that the process of media influence on children may exhibit age differences in the effects of media. Nonetheless, this area has a dearth of research [28,29]. For example, one researcher found no significant relationship between television program viewing and later attention problems in five-year-olds [15]. Therefore, is it possible that young children of different ages show different levels of EF after watching different types of animation for short periods? Answering this question is essential to better understand the sensitive period of influence of animation types on children’s cognitive development. Therefore, this study used an experimental approach to explore the short-term effects of age and animation type on the three components (IC, WM, CF) of four- to seven-year-old children’s EF.

## 2. Materials and Methods

### 2.1. Participants

The number of participants for this experiment was based on previous relevant literature [22,30]. Since the number of participants in each condition in the above studies was 11–15, we aimed to include 12 participants per condition for 144 participants. One hundred fifty-two children from two kindergartens and one first-grade class in an elementary school in a city in Henan Province of China and their parents were included using whole-class sampling. After excluding 26 children whose parents were temporarily absent or unable to complete the questionnaire, 126 participants were validly enrolled. The selected students included 38 four-year-olds (*M =* 49.26 ± 2.50 months; 19 boys and 19 girls); 29 five-year-olds (*M =* 58.34 ± 2.33 months; 17 boys and 12 girls); 32 six-year-olds (*M =* 71.93 ± 2.91 months; 16 boys and 16 girls); and 27 seven-year-olds (*M =* 82.00 ± 3.89 months; 15 boys and 12 girls). The children were randomly allocated to the entertainment group (40), education group (43), or control group (43).

In addition, we conducted a sensitivity power analysis (assumed α = 0.05, power = 0.80). We found that with 12 participants per condition, the minimum effect size for all factors for which we could detect effects (main and interaction) was *f* = 0.30, which was above the medium level of effect (0.25 < *f* < 0.40).

The study protocol followed the American Psychological Association’s ethical guidelines. It was approved by the principals of the kindergarten and primary schools and by the institutional review board of Henan University. All parents provided informed consent. The study complied with the 1964 Helsinki Declaration and its later amendments or comparable ethical standards.

### 2.2. Video Material Screening

#### 2.2.1. Criteria for Video Selection

In the early stage of the study, a questionnaire was used to collect information on the content of animations watched by preschoolers. Based on the cartoon characters, popularity, and reference to the results of previous studies on program selection [20], “Cat and Mouse” (abbreviated as “Cat”) and “Mickey’s Wonderful House” (abbreviated as “Mi”) were selected as the experimental material for this study. This study presented “Mi” for 11 min and 40 s on a screen size of 720 × 576 pixels, referring to previous studies’ criteria for selecting video clip duration [16]. In “Mi,” Mickey and his friends tried to find lost party hats by communicating and collaborating. Each party hat had a different number and color. The color scheme was designed to help children master the colors and numbers. Two episodes of “Cat” (“Mouse Catching Brothers” and “Teaching in the Doctor’s Class”) were selected for editing and synthesis to match with “Mi” in terms of duration. “Cat” lasted 11 min and 10 s, a screen size of 720 × 480 pixels, and a unified compressed audio-video format interleave for the two pieces of material.

The content of the video material was examined for variability by rating according to Zimmerman and Christakis’ criteria for defining educational and entertainment categories [23]. Educational programs included social and cognitive information features, with social information features referring to instruction and teaching content through communication or appropriate behaviors (e.g., solidarity and mutual aid) and cognitive information features referring to teaching content similar to that in school contexts (e.g., other school readiness skills such as counting and comparing sizes). Ten psychology graduate students were required to rate the content of the programs children watched based on the programs’ definitions. Each person provided a score of 0 or 1 to judge the cognitive (10 features) and social information (10 features) characteristics of the video material, with 0 representing the absence of this feature and 1 representing its presence. The total score was considered the video content feature score, with a range of 0–20. By conducting an independent samples *t*-test on the data, the results showed that “Mi” was significantly different from “Cat” in terms of content feature scores (*t* = 14.20, *p* < 0.001), and “Mi” (*M =* 13.90, *SD* = 0.88) had a higher score than “Cat” (*M =* 5.30, *SD* = 1.70). This result indicated that “Cat” met the criteria of entertainment animation and “Mi” met the criteria of educational animation.

#### 2.2.2. Differential Test of Formal Features of Video Material

Considering that formal features of animation may confuse the effect of animation content on EF, it is necessary to match and control the features of video material. Referring to previous studies’ results and measurement criteria [22,31,32], two formal features of video material—pace and fantasy—were selected for evaluation and control. Pace refers to the number of times a complete scene change occurs in the video (e.g., from the pool to the bedroom), and for fast-paced programs, scenes change entirely every 11 s on average [31]. Fantasy refers to transforming objects or characters in impossible ways, such as shape or identity, that are common in our lives but occur against the laws of physics. Ten psychology graduate students rated the pace and fantasy of the video material on a seven-point scale based on the above definitions of complete scene changes per minute and fantasy events per minute. An independent samples *t*-test of the data showed that “Mi” was not significantly different from “Cat” in terms of pace or fantasy score (*t*_pace_ = 1.05, *p* > 0.05; *t*_fantasy_ = 0.45, *p* > 0.05), which indicates that the two types of video material have similar pace and fantasy.

### 2.3. Measures

#### 2.3.1. Parent Questionnaire

Parents completed three questionnaires. The first questionnaire asked for basic information about the family, including family income, parents’ education level, and the sex and age of their children. The second questionnaire comprised information about their child’s exposure to animation, including how much time their child watched animation on weekends and weekdays; the age at which their child first watched animation; and the name of the most recent animation their child watched. A third questionnaire addressed the Childhood Executive Functioning Inventory (CHEXI) as a pre-test indicator of EF. It is ideal to use the same EF task in pre-test and post-test; however, the EF task must involve non-routine stimuli to elicit an appropriate response [33]. When a task is used twice in a short period, it loses its novelty; therefore, we used a parent report scale (CHEXI). This scale is consistent with the EF dimensions of this study and correlates moderately with laboratory EF measures [34,35]. Parents rated each item on a five-point Likert scale. The total score was calculated by averaging the scores of all items, with higher scores indicating more significant EF difficulties. The Cronbach’s alpha in this study was 0.78.

#### 2.3.2. Measurement of EF

This study used a day/night task, a backward digit span task, and the Flexible Item Selection Task to test children’s IC, WM, and CF.

#### 2.3.3. IC

Day-night task: This experimental procedure uses the classic Stroop task [36]. Children are presented with multiple pictures and asked to report “night” when they see a picture with the sun and “day” when they see a picture with the moon. The experiment was divided into two practice trials and 16 formal trials (eight daytime and eight nighttime cards each), and the experimental materials were uniformly presented randomly using slides. Children were scored 0 for incorrect responses and 1 for correct, with a score range of 0 to 16.

#### 2.3.4. WM

Backward digit span task: Referenced from Lillard and Peterson’s digit span task [31], digits 0–9 were randomly combined into groups of two to eight digits. The tester said one digit per second, and the child needed to report them backward. For example, if the tester announces “5-7-4,” the child’s correct report is “4-7-5.” For the practice test, a two-digit sequence of tasks was provided. Once the participant answered one correctly or completed the exercise, they could enter the formal test, and the test was stopped when three sequences were answered incorrectly in a row. Each sequence was repeated three times. A child’s answer was considered correct if they recited the sequence backward one time with no mistakes.

Regarding scoring criteria, the span value of the number sequences correctly recited by the children was recorded. If they could not recite the two-digit sequences correctly, they scored 1 point [37]. The scoring range was 1–8 points.

#### 2.3.5. CF

The Flexible Item Selection Task (FIST): The experimental procedure was based on Jacques and Zelazo’s FIST [38] and Willoughby and Blair’s adaptation of the FIST [39], Something’s the Same Game. The specific measurement process was: (1) Sorting pictures by size, color, and category. (2) Practice phase: first, two pictures with the same dimension were presented, and children were asked to name the dimension. Second, a new picture was presented, and the one that agreed with the new picture in a dimension and that dimension was different from the first two pictures was selected in the first two pictures for three trials. (3) Formal stage: three pictures (containing two different dimensions, e.g., color; size) were presented simultaneously. Two pictures that were identical in one dimension (color) were selected, followed by two identical in the other dimension (size), for 10 trials. The pictures were presented randomly using slides. Regarding scoring criteria, children were given 1 point for the second correct choice, with a score range of 0 to 10.

### 2.4. Experimental Design and Procedures

This experiment had a three (animation type: entertainment, educational, control groups) × four (age: four-, five-, six, and seven-year-olds) between-participants experimental design. The independent variables were the type of animation and the children’s age, and the dependent variable was the EF score. The experiment was administered individually, and children were assigned to different experimental conditions in a Latin square order according to their age. The experimental groups watched different types of animated videos. The control group watched no animations but was provided crayons and paper for the children to draw freely for 11 min.

The experiment was conducted by first leading the child individually into the test room to watch a video (experimental group) or draw freely (control group); the child was then told they would play some mini-games. Second, the same laptop was used to perform the EF tasks for all children; one researcher operated the procedure, and another recorded the children’s task scores. Children then needed only to follow the instructions given by the first researcher to complete the tasks. Finally, the presentation order of the three tasks in the experiment was randomized. At the end of the experiment, children were given a gift worth 10 RMB and were returned safely to the classroom.

### 2.5. Data Statistics and Analysis

A two-step statistical analysis of the data was performed using SPSS 20.0. In the first step, pre-test analyses were conducted using multivariate analysis of variance (MANOVA) to analyze differences in pre-test variables among the three groups of children with animation type and post-hoc tests using the Bonferroni test. Formal analysis was conducted in the second step, using the three components of EF as dependent variables and age and animation type as independent variables. A MANOVA was conducted using the significant variables from the first step as control variables, and Bonferroni tests were used to test for differences in main and interaction effects. Significance was defined as a *p*-value < 0.05.

## 3. Results

### 3.1. Pre-Test Results

Table 1 shows the descriptive statistics of the three groups of children on each background variable. The results of the ANOVA revealed that the three groups of children had a non-significant difference in sex (*F*_(2,123)_ = 0.69, *p =* 0.50), family income (*F*_(2,123)_ = 0.35, *p =* 0.70), mother’s education level (*F*_(2,123)_ = 1.55, *p =* 0.22), age of first exposure to animation (*F*_(2,123)_ = 0.07, *p =* 0.93), hours of animation watched on weekdays (*F*_(2,123)_ = 0.93, *p =* 0.40), hours of animation watched on weekends (*F*_(2,123)_ = 2.97, *p =* 0.06), and pre-test EF score (*F*_(2,123)_ = 0.03, *p =* 0.97) were non-significant. Therefore, these variables were not considered in further analyses.

### 3.2. Short-Term Effects of Animation Type on EF Components in Children

Table 2 shows the task scores of the three EF components in each group. Data were analyzed using a three (type of animation) × four (age) MANOVA. The analysis found significant main effects for group (Pillai’s trace = 0.25, *F*_(6,226)_ = 5.45, *p* < 0.001, η^2^ = 0.13) and age (Pillai’s trace = 0.53, *F*_(9,342)_ = 8.06, *p* < 0.001, η^2^ = 0.18), while the interaction effect of group and age was non-significant (Pillai’s trace = 0.15, *F*_(18,342)_ = 1.00, *p =* 0.46, η^2^ = 0.05). The EF components with significant main effects were further analyzed, and the results are as follows.

#### 3.2.1. Effect of Animation Type on Children’s IC

**Figure 1:**
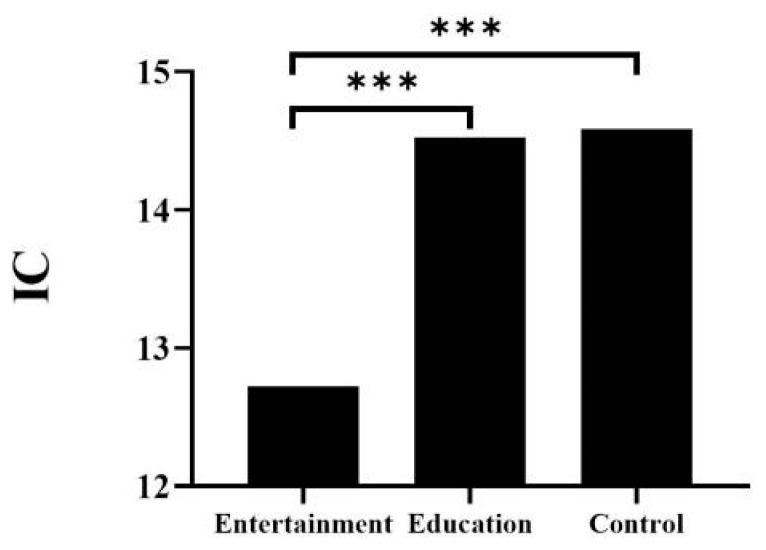
Comparison of IC in different groups. *** *p* < 0.001; IC: inhibitory control.

For IC, the main effect of age was significant (*F*_(3,114)_ = 28.93, *p* < 0.001. η^2^ = 0.43). Post-hoc Bonferroni tests showed that the scores of the four- and five-year-old groups were significantly lower than those of the seven-year-old group (*p* < 0.001), and the other differences between the groups were non-significant. The main effect of animation type was also significant (*F*_(2,114)_ = 13.22, *p* < 0.001, η^2^ = 0.96). Post-hoc Bonferroni tests showed that children in the entertainment group scored significantly lower for IC than those in the educational and control groups (*p* < 0.001). There was no difference in IC between the educational and control groups (*p* > 0.05; Figure 1).

#### 3.2.2. Effect of Animation Type on Children’s WM

For WM, the main effect of age was significant (*F*_(3,114)_ = 28.93, *p* < 0.001, η^2^ = 0.43). Post-hoc Bonferroni tests showed that the scores of the four-year-old group were significantly lower than those of the other three age groups (*p* < 0.001); the scores of the five- and six-year-old groups were significantly lower than those of the seven-year-old group (*p* < 0.001); and the difference between the five- and seven-year-old groups was non-significant. There was no significant main effect of the type of animation (*F*_(2,114)_ = 2.63, *p >* 0.05).

#### 3.2.3. Effect of Animation Type on Children’s CF

**Figure 2:**
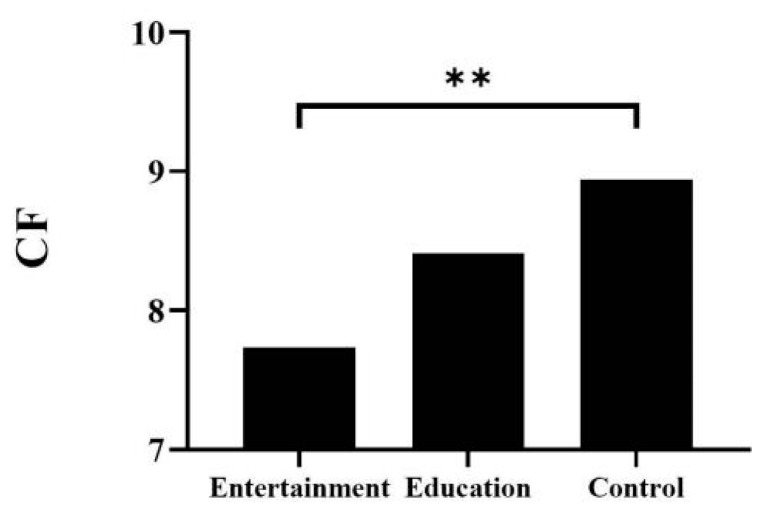
Comparison of CF in different groups. ** *p* < 0.01; CF: cognitive flexibility.

For CF, the main effect of age was significant (*F*_(3,114)_ = 18.05, *p* < 0.001, η^2^ = 0.18). The post-hoc Bonferroni test indicated that the scores of the four-, five-, and six-year-old groups were significantly lower than those of the seven-year-old group (*p* < 0.001), and the difference between the other groups was non-significant (*p* > 0.05). The main effect of the animation type was significant (*F*_(2,114)_ = 4.66, *p* = 0.01, η^2^ = 0.08). The post-hoc Bonferroni test showed that children’s CF scores were significantly lower in the entertainment group than in the control group (*p* < 0.01) and that the other differences between the groups were non-significant (*p* > 0.05; Figure 2).

## 4. Discussion

This study examined the short-term effects of animations on EF tasks in children aged four to seven years, and explored the moderating role of age in this relationship. The results found that short-time viewing of different types of animation affected children’s subsequent performance on EF tasks; viewing entertainment-type animation reduced children’s IC and CF scores, while no effects were found for educational-type animation on each EF task. Furthermore, these effects were the same across ages.

### 4.1. Short-Term Effects of Animation Type on EF in Children

In the current study, animation type affected children’s IC tasks: entertainment animation weakened children’s IC scores more than educational animation or drawing. The main features of entertainment programs are lively storylines with robust narrative features. These features attract children’s attention and stimulate their interest [31,40]. Li’s study assessed children’s eye movements while watching different types of programs. The results showed that entertainment programs elicited shorter gaze duration and more gaze points than educational programs [20]. Thus, fun-rich entertainment programs elicit great interest from children.

Moreover, frequent transitions in program plots or scenes increase the demand for children’s encoding and storage resources [41]. According to the strength model of self-control, all self-control requires the same resources, and the consumption of resources in one domain affects the resources available in another [42]. In addition, the cognitive resources consumed by entertainment animation reduce the resources available to inhibit certain behaviors and thus result in a poorer response in corresponding tasks.

Furthermore, the most significant difference between entertainment and educational programming is that entertainment programs lack the cognitive and social information features that enhance delayed tolerance in young children [18] or are harmless to children’s cognitive development [23]. Thus, entertainment programs’ absence of informational features may place children in a passive mode of information processing when watching the program [43] and perform worse on IC tasks. Therefore, considering that IC is the basis for the development of complex cognitive functions in children, a concerted effort by parents and society to reduce the viewing of entertaining animation by children before age seven is warranted to promote their healthy cognitive development.

Entertainment cartoons may weaken CF due to limited cognitive resources and the characteristics of CF. First, according to the strength model of self-control [42], children must constantly suppress existing real-world rules and knowledge when watching entertainment cartoons and conform to illusory plots while consuming their modest cognitive resources. Second, according to Lang’s limited capacity model [41], animation’s distinctive and prominent features attract children’s attention. This result leads to a constant response to the frequent and novel stimuli of the program, raising the level of individual arousal and increasing children’s need for information encoding. However, children’s limited cognitive resources cannot meet these new demands, especially as more complex CF may require IC and WM together [44], thus requiring more resources to process the information. Therefore, the appropriation of limited cognitive resources cannot satisfy tasks that subsequently require the involvement of more resources. This shift causes individuals to exhibit diminished CF. Hence, practical steps are needed to promote children’s CF and reduce cognitive attrition. Therefore, reducing the cartoon watching time and frequency for children aged four to seven years is essential. In addition, parents and educators should encourage them to spend more time on cognitively beneficial activities such as drawing or sports.

Finally, this study found no significant differences in children’s WM levels between the drawing and the entertainment and educational animation groups. This finding may be related to the measurement tool used in the present study. The present study used a backward digit span task. Although trends in this task were also found across ages in this research, considering this task is a complex memory span task [45], its critical developmental period would be in elementary school [46]. Thus, the participants in the present study were probably only at the low level of the task (the mean value of the subjects in the present study on this task was only 2.69). The low differentiation on the task prevents the effect of animation from being well-differentiated. Future validation studies could be conducted using simple WM tasks or visual WM tasks that are more applicable to the early childhood stage.

### 4.2. Age Differences in the Effects of Animation on Children’s EF

Although the present study found significant age differences in EF scores for each task, that is, there was a tendency for scores for each component of EF to improve with increasing age. However, there was no significant age difference in the effect of animation type on children’s EF. This finding may be attributed to the developmental stage of EF. Although there are differences in the development of EF components in children aged four to seven, these children are all in the initial developmental stage of EF [47], so animation’s effects on EF are similar for all these ages. Alternatively, the results may indicate that the age differences in animation effects on EF in children are not observable between four and seven years. Since infancy is a period of rapid brain development and plasticity, television may impair EF more severely during that time than during other developmental periods [12].

Furthermore, there is no meaningful relationship between television viewing and attention problems at five years old [15]. However, a significant relationship exists between television viewing at age one and later attention problems [10]. Combined with the American Academy of Pediatrics’ suggested ban on video media for children under the age of two [48], it is more likely that the sensitive period for media effects on children’s EF is before rather than after four years of age. Future studies should investigate these inferences.

### 4.3. Limitations

Although this study yielded some meaningful results, there are limitations. First, drawing was used as a control group when grouping animation types. Although drawing is a quiet activity often performed by young children and is often used by control groups, this activity may promote children’s EF to some extent rather than not affect EF. Therefore, an activity with no effect on EF is needed to reveal the actual effect of animation type on EF objectively. Second, although a causal conclusion can be drawn from the short-term effect of animation on children’s EF, this may only be temporary, and there may not be a long-term effect on children’s EF. Therefore, long-term cumulative effects should be investigated in future research. Third, while this study controlled for features such as fantasy and pacing in selecting animation, there is a complex combination of these animation features and types (educational vs. entertainment) in everyday animation. Future research could build on this foundation and explore the effects of different combinations of animation messages on children to understand better the effects of animation on children’s EF. Finally, this study only applied three widely used tasks to measure the three components of EF, which only reflected a specific characteristic of each component of EF and could not represent the connotation of each component more comprehensively. Future studies can consider different connotations of EF tasks concurrently and select multiple tasks for comprehensive consideration.

## 5. Conclusions

Viewing cartoons impairs subsequent EF performance in children aged four to seven years, as evidenced by the fact that viewing entertainment animation impairs children’s IC and CF, whereas viewing educational animations did not affect children’s performance on each task of EF. Entertainment animation viewing behavior still needs to be limited and reduced for children younger than seven years.

## Figures and Tables

**Table 1:** Descriptive statistics of the three groups of children for each variable.

Variable	Entertainment	Educational	Control	Value Range
Sex	0.50 ± 0.51	0.49 ± 0.51	0.60 ± 0.49	[0.00, 1.00]
Family monthly income (RMB)	3.46 ± 1.08	3.65 ± 1.00	3.53 ± 1.07	[1.00, 5.00]
Mother’s education	3.10 ± 1.15	3.49 ± 0.98	3.26 ± 0.90	[1.00, 5.00]
Age of first exposure to animation (months)	26.23 ± 11.66	25.30 ± 10.91	25.49 ± 11.98	[3.00, 60.00]
Animations viewed on weekdays (hours per day)	1.82 ± 0.96	2.02 ± 1.06	2.12 ± 0.96	[0.00, 4.00]
Animations viewed on weekends (hours per day)	1.67 ± 1.05	2.02 ± 1.04	2.23 ± 1.07	[0.00, 4.00]
Pre-EF score	1.76 ± 0.34	1.77 ± 0.44	1.78 ± 0.27	[1.13, 3.79]

Sex: 0 = female, 1 = male. Family monthly income: 1 = <3000 yuan, 2 = 3000 to 5000 yuan, 3 = 5000 to 7000 yuan, 4 = 7000 to 9000 yuan, and 5 = >9000 yuan. Mother’s education: 1 = <junior high school, 2 = high school or junior college, 3 = college; 4 = bachelor’s degree, 5 = postgraduate.

**Table 2:** Scores of each EF component for different groups.

Age (Years)	Inhibitory Control			Working Memory			Cognitive Flexibility		
	Entertainment	Educational	Control	Entertainment	Educational	Control	Entertainment	Educational	Control
Four	12.21 ± 2.33	13.40 ± 1.50	14.00 ± 1.41	2.43 ± 0.65	3.07 ± 0.88	2.45 ± 0.82	6.71 ± 2.43	7.80 ± 1.57	8.55 ± 1.29
Five	12.50 ± 3.17	14.44 ± 1.42	13.67 ± 1.30	3.50 ± 0.97	3.56 ± 0.73	3.42 ± 0.90	7.70 ± 1.57	7.67 ± 1.50	8.25 ± 2.83
Six	12.57 ± 3.03	14.83 ± 1.47	15.18 ± 0.98	3.57 ± 0.94	3.92 ± 0.79	4.00 ± 0.63	7.29 ± 1.98	8.00 ± 2.34	9.45 ± 0.93
Seven	14.62 ± 1.26	15.07 ± 1.10	15.56 ± 0.63	4.46 ± 1.06	4.53 ± 0.83	4.94 ± 0.85	9.54 ± 0.78	9.67 ± 0.82	9.56 ± 0.81

## Data Availability

The data presented in this study are available on request from the corresponding author.

## References

[1] Beck D.M., Schaefer C., Pang K., Carlson S.M. (2011). Executive function in pre-school children: Test-retest reliability. J. Cogn. Dev..

[2] Diamond A. (2013). Executive functions. Ann. Rev. Psychol..

[3] Kim S., Nordling J.K., Yoon J.E., Boldt L.J., Kochanska G. (2013). Effortful control in “hot” and “cool” tasks differentially predicts children’s behavior problems and academic performance. J. Abnorm Child. Psychol..

[4] Ponitz C.C., McClelland M.M., Matthews J.S., Morrison F.J. (2009). A structured observation of behavioral self-regulation and its contribution to kindergarten outcomes. Dev. Psychol..

[5] Moffitt T.E., Arseneault L., Belsky D., Dickson N., Hancox R.J., Harrington H., Houts R., Poulton R., Roberts B.W., Ross S. (2011). gradient of childhood self-control predicts health, wealth, and public safety. Proc. Natl. Acad. Sci. USA.

[6] Bernier A., Carlson S.M., Deschênes M., Matte-Gagné C. (2012). Social factors in the development of early executive functioning: A closer look at the caregiving environment. Dev. Sci..

[7] Rideout V. (2011). Zero to Eight: Children’s Media Use in Americ.

[8] Tandon P.S., Zhou C., Lozano P., Christakis D.A. (2011). Preschoolers’ total daily screen time at home and by type of child care. J. Pediatr..

[9] Dong S., Song Y., Jiang Y., Sun G., Wang Y., Jiang F. (2015). A multicenter study on the effect of television viewing behavior on sleep quality in children under 4 years old in China. Chin. J. Pediatr..

[10] Christakis D.A., Zimmerman F.J., Di Giuseppe D.L., McCarty C.A. (2004). Early television exposure and subsequent attentional problems in children. Pediatrics.

[11] Miller C.J., Marks D.J., Miller S.R., Berwid O.G., Kera E.C., Santra A., Halperin J.M. (2007). Brief report: Television viewing and risk for attention problems in pre-school children. J. Pediatr. Psychol..

[12] Nathanson A.I., Aladé F., Sharp M.L., Rasmussen E.E., Christy K. (2014). The relation between television exposure and executive function among preschoolers. Dev. Psychol..

[13] Parkes A., Sweeting H., Wight D., Henderson M. (2013). Do television and electronic games predict children’s psychosocial adjustment? Longitudinal research using the UK Millennium Cohort Study. Arch. Dis. Child..

[14] Foster E.M., Watkins S. (2010). The value of reanalysis: TV viewing and attention problems. Child. Dev..

[15] Stevens T., Muslow M. (2006). There is no meaningful relationship between television exposure and symptoms of attention-deficit/hyperactivity disorder. Pediatrics.

[16] Anderson D.R., Hanson K.G. (2009). Children, media, and methodology. Am. Behav. Sci..

[17] Calvert S.L., Wilson B.J. (2008). Media Violence and Aggression in Youth.

[18] Friedrich L.K., Stein A.H. (1973). Aggressive and prosocial television programs and the natural behavior of preschool children. Monogr. Soc. Res. Child. Dev..

[19] Geist E.A., Gibson M. (2000). The effect of network and public television programs on four and five year olds ability to attend to educational tasks. J. Instr. Psychol..

[20] Li H. (2014). The Impact of Television on Young Children’s Executive Functioning: A Perspective on Authenticity Judgments. Doctoral Dissertation.

[21] Yang X., Chen Z., Wang Z., Zhu L. (2017). The relations between television exposure and executive function in Chinese preschoolers: The moderated role of parental mediation behaviors. Front. Psychol..

[22] Lillard A.S., Drell M.B., Richey E.M., Boguszewski K., Smith E.D. (2015). Further examination of the immediate impact of television on children’s executive function. Dev. Psychol..

[23] Zimmerman F.J., Christakis D.A. (2007). Associations between content types of early media exposure and subsequent attentional problems. Pediatrics.

[24] Anderson V., Levin H.S., Jacobs R., Stuss D.T., Knight R.T. (2002). Executive functions after frontal lobe injury: A developmental perspective. Principles of Frontal Lobe Function.

[25] Brookshire B., Levin H.S., Song J., Zhang L. (2004). Components of executive function in typically developing and head-injured children. Dev. Neuropsychol..

[26] Wiebe S.A., Espy K.A., Charak D. (2008). Using confirmatory factor analysis to understand executive control in pre-school children: I. Latent structure. Dev. Psychol..

[27] Wilson J., Andrews G., Hogan C., Wang S., Shum D.H.K. (2018). Executive function in middle childhood and the relationship with theory of mind. Dev. Neuropsychol..

[28] Valkenburg P.M., Peter J. (2013). The differential susceptibility to media effects model. J. Commun..

[29] Beyens I., Valkenburg P.M., Piotrowski J.T. (2018). Screen media use and ADHD-related behaviors: Four decades of research. Proc. Natl. Acad. Sci. USA.

[30] Cooper N.R., Uller C., Pettifer J., Stolc F.C. (2009). Conditioning attentional skills: Examining the effects of the pace of television editing on children’s attention. Act. Paediatr..

[31] Lillard A.S., Peterson J. (2011). The immediate impact of different types of television on young children’s executive function. Pediatrics.

[32] Xing S., Jiang Y., Gao X., Ding B., Yang Y. (2017). Long-term effects of television on pre-school children’s executive function development: An empirical study. Educ. Res..

[33] Müller U., Kerns K., Liben L.S., Müller U., Lerner R.M. (2015). The development of executive function. Handbook of Child Psychology and Developmental Science, Vol. 2: Cognitive Processes.

[34] Thorell L.B., Nyberg L. (2008). The Childhood Executive Functioning Inventory (CHEXI): A new rating instrument for parents and teachers. Dev. Neuropsychol..

[35] Camerota M., Willoughby M.T., Kuhn L.J., Blair C.B. (2018). The Childhood Executive Functioning Inventory (CHEXI): Factor structure, measurement invariance, and correlates in US preschoolers. Child. Neuropsychol..

[36] Gerstadt C.L., Hong Y.J., Diamond A. (1994). The relationship between cognition and action: Performance of children 3 1/2–7 years old on a Stroop-like day-night test. Cognition.

[37] Carlson S.M., Moses L.J., Breton C. (2002). How specific is the relation between executive function and theory of mind? Contributions of inhibitory control and working memory. Infant Child. Dev..

[38] Jacques S., Zelazo P.D. (2001). The flexible item selection task (fist): A measure of executive function in preschoolers. Dev. Neuropsychol..

[39] Willoughby M.T., Blair C.B. (2016). Measuring executive function in early childhood: A case for formative measurement. Psychol. Assess..

[40] Goodrich S.A., Pempek T.A., Calvert S.L. (2009). Formal production features of infant and toddler DVDs. Arch. Pediatr. Adolesc. Med..

[41] Lang A. (2000). The limited capacity model of mediated message processing. J. Commun..

[42] Baumeister R., Vohs K., Tice D.M. (2007). The strength model of self-control. Curr. Dir. Psychol. Sci..

[43] Huston A.C., Wright J.C., Bryant J., Anderson D.R., Bryant J., Anderson D.R. (1983). Children’s processing of television: The informative functions of formal features. Children’s Understanding of Television: Research on Attention and Comprehension.

[44] Best J.R., Miller P.H. (2010). A developmental perspective on executive function. Child. Dev..

[45] Gathercole S.E., Pickering S.J., Ambridge B., Wearing H. (2004). The structure of working memory from 4 to 15 years of age. Dev. Psychol..

[46] Demetriou A., Spanoudis G., Makris N., Golino H., Kazi S. (2021). Developmental reconstruction of cognitive ability: Interactions between executive, cognizance, and reasoning processes in childhood. Cog. Dev..

[47] Tu M., Yang F. (2018). Progress of foreign research on executive functions of pre-school children in the last decade. Preschool. Educ. Res..

[48] American Academy of Pediatrics (2013). Children, adolescents, and the media. Pediatrics.

